# Selling second best: how infant formula marketing works

**DOI:** 10.1186/s12992-020-00597-w

**Published:** 2020-08-28

**Authors:** Gerard Hastings, Kathryn Angus, Douglas Eadie, Kate Hunt

**Affiliations:** 1 Institute for Social Marketing and Health, University of Stirling, Stirling, FK9 4LA Scotland, UK; 2 L’École des Hautes Études en Santé Publique, Rennes, France

**Keywords:** Commercial determinants of ill-health, Infant formula, Breast milk substitutes, Marketing, Multinational corporations, Corporate power

## Abstract

**Background:**

Despite the clear policy intent to contain it, the marketing of formula milk remains widespread, powerful and successful. This paper examines how it works.

**Methods:**

The study comprised a mix of secondary analysis of business databases and qualitative interviews with marketing practitioners, some of whom had previously worked in formula marketing.

**Results:**

The World Health Assembly Code aims to shield parents from unfair commercial pressures by stopping the inappropriate promotion of infant formula. In reality marketing remains widespread because some countries (e.g. the USA) have not adopted the Code, and elsewhere industry has developed follow-on and specialist milks with which they promote formula by proxy. The World Health Assembly has tried to close these loopholes by extending its Code to these products; but the marketing continues. The campaigns use emotional appeals to reach out to and build relationships with parents and especially mothers. Evocative brands give these approaches a human face. The advent of social media has made it easier to pose as the friend and supporter of parents; it is also providing companies with a rich stream of personal data with which they hone and target their campaigns.

The formula industry is dominated by a small number of extremely powerful multinational corporations with the resources to buy the best global marketing expertise. Like all corporations they are governed by the fiduciary imperative which puts the pursuit of profits ahead of all other concerns. This mix of fiscal power, sophisticated marketing, and single-mindedness is causing great harm to public health.

**Conclusions:**

Formula marketing is widespread and using powerful emotional techniques to sell parents a product that is vastly inferior to breast milk. There is an urgent need to update and strengthen regulation.

## Background

The commercial determinants of ill-health are now well recognised. In particular, many of the products we consume – tobacco, processed food, alcohol, petrochemicals, leaded paint, guns – are known to have caused such harm, even when used as intended, that a new descriptor, the ‘industrial epidemic’ [1], has been coined. Whilst free choice and consumer sovereignty are much lauded, in reality this destructive consumption behaviour is not altogether voluntary; we are energetically encouraged to smoke, drive cars and arm ourselves by those who gain from our self-harm - the companies that make and sell these products. In recent years these industries have grown in size, led by multinational corporations with powerful lobbying and corporate affairs functions with which to engage policy makers. So, soda makers can influence the Centers for Disease Control and Prevention [2], oil companies undermine climate science [3] and the paint industry exonerate lead [4], and in the process regulation is avoided, delayed or contained.

This ensures an environment where marketing – the persuasive tool of choice for consumers – can be used with maximum efficiency. Its capacity to encourage consumption has been established in multiple studies for tobacco, alcohol and processed food [5–7]. The methods used by marketers have also been examined, and the role of emotional appeals, branding and careful targeting noted [5]. The advent of digital technologies has raised further concerns about the insidious power of social media marketing, and the bespoke, deep messaging it facilitates. Facebook gets over 98% of its income from advertising [8], and the Cambridge Analytica revelations show how pervasive and profound digital influence has become [9].

This paper concerns an industrial epidemic that has been going on for four decades in the infant feeding domain: the breast milk substitutes (BMS) industry. A recent analysis shows that if all babies were breastfed as the World Health Organization (WHO) recommends, over 800,000 infant deaths would be avoided each year [10]. BMS also harm the intellectual development of the baby to such an extent that it is possible to detect the impact on GDP of a predominantly bottle-fed population [11]. In addition, there is an increased risk of breast cancer for the mother [10], and significant ecological harm: packaging, supply chains and bottle-feeding apparatus all have a carbon footprint and introduce durable plastics into the environment [11]. The competition, breast milk, has none of these drawbacks, and comes with natural antibodies that turn it into “a personalised medicine for infants” [10]; manufactured products cannot begin to replicate these benefits. Breast milk is also much cheaper. Bottle feeding a baby for six months in the UK today costs £175 (approx. US$210) for the cheapest own-label products, and more than double that for a premium brand [12], and these figures do not include any equipment, such as bottles, teats and sterilisers. Comparable data from the USA suggest that it could cost between US$451 and $810 to buy BMS product to feed a baby for six months [13]. There are some advantages to bottle-feeding: for some women, breastfeeding can be difficult to instigate and maintain so formula is a necessary alternative; conflicts can arise (for mothers or observers) between the feeding and sexual functions of the breast; and unsupportive public and work places make breastfeeding difficult. The fact that breastfeeding is not readily accommodated by the world of work worldwide makes it particularly challenging for women to breastfeed in the absence of or beyond any period of maternity leave. This is a marked problem in low-income countries where welfare systems are less well-developed. However, in most cases, when taken in the context of threats to the baby’s life and future prospects, or the risk of cancer, these benefits become much less persuasive.

Selling a product which falls so far behind the competition seems challenging, but the evidence shows it can and is being done with remarkable success. Marketing has, as with other health harming products, been proven to encourage formula consumption [14] and sales are increasing 8% year-on-year; the global market for BMS products was forecast to reach US$70.7 billion by 2019 [11]. Across the world, in high- and low-income countries alike, only 40% of mothers now follow WHO breastfeeding guidelines [15].

Concerns about the marketing of infant formula are not new. Forty years ago, the World Health Assembly (WHA; the decision-making body of the world’s Member States) developed its International Code of Marketing of Breast-milk Substitutes [16] which required companies to acknowledge the superiority of breast milk, and outlawed any advertising or promotion of BMS to the general public. In reality, marketing remains widespread because some countries (e.g. the USA) have not adopted the Code and elsewhere industry has developed follow-on and specialist milks which they use to promote infant formula by proxy – that is, they are branded in exactly the same way and the boundaries between infant formula and follow-on and other products are blurred. The WHA has moved to close these loopholes by clarifying that the Code also applies to these products [17]; but the marketing continues. The advent and proliferation of digital media has further undermined the Code.

This study was designed to understand how BMS formula marketers have succeeded, despite the known inferiority of their product in comparison with breast milk and the exigencies of the Code. Their methods have previously been audited and described [16, 18–22]; we aimed to analyse and explain them.

## Methods

We used a mixed methods approach involving two linked data collection exercises: a review of publicly available data on the global marketing of breast milk substitutes, followed by qualitative interviews with marketing practitioners with experience of breast milk substitutes and food marketing.

### Marketing and business literature review

The marketing review was designed to identify documents and data to describe the scale of the global breast milk substitutes market, including current forecasting of future directions and priorities of marketing strategies; to identify marketing and brand strategies; and to inform the qualitative interviews.

A range of search techniques, including snowballing, was used to identify relevant material. Subscription business, academic and practitioner databases were searched in April–May 2019: Business Source Complete, IBISWorld, Marketline Industry Profiles, Nexis, SAGE Business Cases, Statista and WARC (World Advertising Research Center). Example search terms included: babies formula, baby formula, baby nutrition, bottle feeding, breast milk, breastmilk, follow-on milk, infant formula, milk powder, milkpowder, mother’s milk substitute, mother’s milk, powdered formula, powdered milk, toddler formula, toddler milk. A generic search engine and the reference lists and bibliographies of relevant reports were used to identify further market research intelligence reports and global marketing materials. Websites of the two BMS producers with the largest global market share [18] were searched for reports and information for shareholders, as well as marketing examples for their brands.

The analysis of documents and secondary data was not intended to be comprehensive; rather we aimed to update accessible data on the scale of the BMS market and provide illustrative business and marketing strategies. The documents and data often referred to the much broader categories of baby and infant food and nutrition, thus market and marketing data for the BMS category were limited, and market research intelligence reports are still prohibitively expensive [21]. Identified documents were carefully scrutinised and all relevant data extracted by one author (KA) into a project resource file. The extracted data were organised by type (market size and forecast, marketing budgets, marketing strategies and techniques) for reporting findings. Noteworthy data and brand case studies were shared among the article authors for further analysis and use in the qualitative interviews.

### Qualitative interviews

The qualitative interviews were designed to examine: how manufacturers of breast milk substitutes position themselves and their products to compete against breast milk, using a marketing framework; and how these strategies and approaches are likely to evolve in the future. The aim was to conduct a series of ~ 6–8 semi-structured interviews with industry experts and professionals with experience of marketing BMS and other commercial food products who were willing to talk candidly about their views and experiences. These included independent marketing consultants, communications specialists and industry insiders, and those with experience in both high and low income countries. These interviews also involved the collection of case materials and written responses and were supported by additional contextual interviews (up to ~ 10) with breast milk and breastfeeding advocates who provided information and advice on accessing industry informants.

All interviews were conducted either face-to-face or by telephone, typically lasted 90–120 min and were guided using an interview schedule which was deliberately loosely structured to enable participants to talk flexibly and freely about their experiences. Interviews examined the full breadth of marketing variables (product, price, promotion and place, commonly referred to as the 4Ps) and how these are used to develop brands and brand families. Links to consumer behaviour were examined in detail. Participants were also encouraged to discuss how BMS are currently being marketed and how this may change or develop in the future, especially online and in relation to digital marketing. Examples of existing marketing materials generated from the literature review were used as prompts to help stimulate discussion.

Participants were purposively selected through existing professional and academic networks using a combination of cascading techniques and personal recommendation, and relied on a combination of face-to-face, telephone and email communication, along with support from breast milk and breastfeeding advocates. Prospective candidates were emailed a copy of the study participant information sheet and consent form and followed up by telephone and/or email as required. Where appropriate, commercial participants were offered a fixed cash incentive as a gesture of thanks for their time and a contribution to any costs of taking part. All participants who expressed a wish to take part were asked to provide informed consent, either verbally or in writing. Given the sensitive nature of the topic area all participants were offered full anonymity as part of the conditions for taking part. All interviews were conducted by two of the authors (GH, DE), and for the most part with individual respondents; in one instance two participants were interviewed together. A total of 26 individuals were approached for an interview from the UK, Continental Europe, North America, Australia and New Zealand, with 20 participants agreeing to take part. These included BMS industry representatives with experience in formula milk marketing and product development (*n* = 6), communications and market research consultants with experience in food and social marketing (*n* = 10), public health experts (PHE; *n* = 2) and breastfeeding advocates (*n =* 2). All of the interviews were completed between January and June 2019 and were conducted in English with one exception (French); one participant also provided a follow-up interview.

All interviews were recorded on digital voice-file with participants’ consent and then professionally transcribed and archived using non-identifiable codes prior to analysis. Given the small number of interviews involved, analysis was conducted manually by the two authors responsible for conducting the interviews, led by GH. The transcripts were reread repeatedly to identify emerging themes and the reliability of these themes reassessed by a process of cross-examination with any interpretative differences resolved through discussion. These analyses allowed the investigation team to identify patterns across the data as a whole and to draw iterative comparisons.

### Data synthesis

Data from the two research strands were reviewed by all authors for common themes and explanations for how the BMS marketing works and factors which contribute to and help explain its success.

Quotes from interviewees and the marketing documents and case studies illustrate the explanations of the marketing techniques used: consumer research, relationship building, segmentation and targeting, stakeholder marketing and promotional appeals. The market data, budgets and strategies then contextualise these findings.

## Results

Formula marketing, as for other fast-moving consumer goods, starts with a detailed understanding of the customer; on this can be built long-term relationships which are strengthened with careful segmentation and targeting. The resulting campaigns work at both a brand and generic level. Maintaining stakeholder support is also important. The fiscal strength of the key players ensure that this marketing activity is guided by the best global expertise.

The quotations in the results section come primarily from the interviewees with direct experience of BMS marketing (formula marketing experts [FMEs]), but the sentiments expressed reflect the comments of all the marketing experts interviewed. In addition, references are made to marketing and business documents, and in these cases citations are included.

### Understanding your customer

**Fig. 1:**
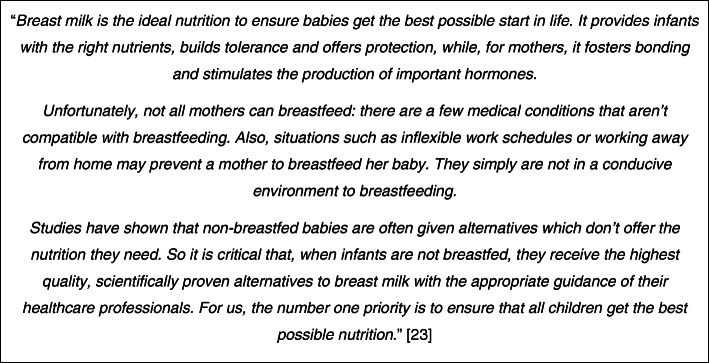
Breast is best, but [23]

Marketing is a complex and sophisticated art. In the formula industry, as in other consumer goods sectors, marketers seek to solve their customers’ ‘problems’, and to do so effectively it is essential to gain a detailed understanding of *“who are you talking to, what’s in their head, how can you engage them, how do you sell* *yourself* *to that person*” (FME). The approach is indirect, very much “*a soft sell”* building faux-friendships rather than making an overt sales pitch: *“we want to build a relationship with you as a mother, we want to support you, we want you to see us as an ally and we want to subtly insinuate ourselves as your friend and support in a healthy pregnancy and a happy baby”* (FME)*.* Paradoxically, the only unmistakably factual material that is always included is the ‘breast is best’ declaration required by the WHO Code, but this is also used to good marketing effect. First, it aligns the company with WHO and the public health establishment. Second, it raises the topic of “*first milk”*, which is supposed to be a no-go area for marketing: “*they cannot legally communicate about the first milk, it’s legally forbidden in most of the countries so they are always playing with the …* [requirement to] *mention that it is the best thing* *after* *the maternal milk”* (FME)*.* For example, one company’s ‘breast is best’ statement continues *“unfortunately, not all mothers can breastfeed …*”*,* and so into an overt pitch for its products (see Fig. 1).

Third it helps maintain a pretence that formula milk does not compete with breast milk: *“so they are not even competing … they are smarter than that, they are just saying yeah, yeah of course the milk from the woman is the best, right, however we bring you this, this and this”* (FME)*.* At the same time, the commercial realities are clear: *“in the ‘Baby Book’ they do track the percentage of what they call ‘share of stomach’, that is breast milk, so they are aware of rates of breastfeeding in a country, but I never saw any documents or strategies anywhere that were about how to get women away from breastfeeding. I mean it must be. Surely, it’s in the back of their minds; this is the free alternative that is reducing their market share, but there wasn’t conscious recognition of that so maybe that’s political I don’t know. Maybe the most senior people, they do talk about it”* (FME).

### Building long-term relationships with baby clubs and carelines

The relationship-building, its nuance and subtlety notwithstanding, is equally strategic; well-established relationships will last for years: *“*[corporation name] *is always on a quest to find ways to identify women who are pregnant for the first time … right when they find out they are pregnant or early in their pregnancy because … how a woman feeds her first baby is how she is likely to feed her subsequent babies … first time mothers are the holy grail”* (FME)*.* Relationships can also span generations: *“the music of* [brand name]*, Baby Love* [by The Supremes], *has been there for more than forty years … so imagine your mum heard it, now you are hearing it; that’s an iconic asset; it’s running through generations”* (FME)*.*

**Fig. 2:**
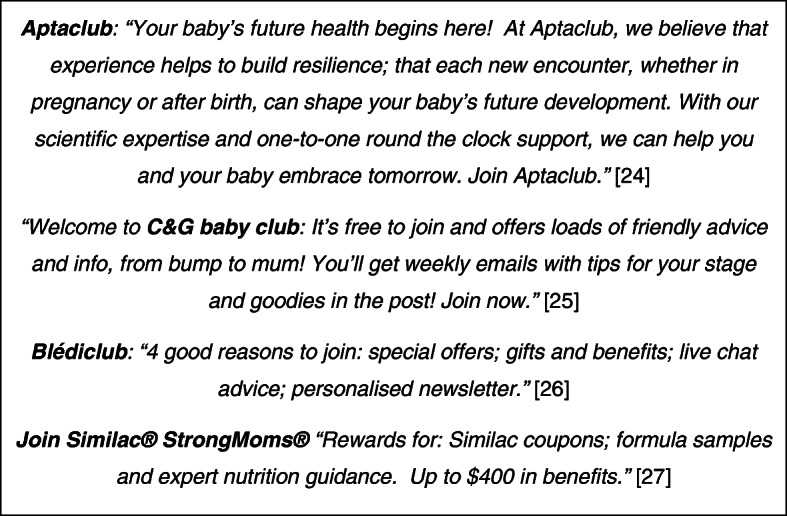
Baby Clubs [24–27]

Baby ‘clubs’ (Fig. 2) and telephone advice lines are the favoured vehicles for establishing and fostering these relationships: *“we had a particular focus on what they call ‘one to one marketing’ which is reaching mothers individually and building individual relationships with mothers. The two big tools in their arsenal, their two favourite tools, were the* [telephone advice line] *and the* [baby club] *… I’d spend a lot of time advising marketing teams* [in different countries] *that the first two things you do are set up your* [baby club and telephone advice line]*; you can then do other things, but those are the two direct relationship building* [tools] *with mothers; those are the two ace cards to play”.* And it is still very much a soft sell: *“there is no mention of formula on the* [telephone advice line], *it’s just about insinuating the products as your friend”*.

Digital technology has greatly enhanced these tools: *“I have decks and decks of the different apps and digital things that* [corporation name] *created. Basically, their process was to ask in a given country what kinds of mothers are we talking to, what are the needs that those mothers have, and therefore what digital marketing do we create to meet those needs”* (FME). These apps range from *“an online ovulation calculator, to help women get pregnant in the first place”* to *“an app for mothers to reach other mothers who were up all night, so mothers who have a newborn baby and they are up at three am and they are lonely and bored, could connect to other mothers who are up at the same time and have a chat”* (FME). Similarly, “*when you sign up to the* [baby club] *you tell them what your due date is and whether you are at two months or eight months or wherever you are, and then you step into a series of emails that are timed to your stage of pregnancy”* (FME)*.* In return for this targeted support, the company gets a constant stream of personal data as well as enhanced sales: “*they had significant evidence to show that these are effective at driving sales literally … they had very good evidence to show that if a woman is in the* [baby club]*, if a woman has called the* [telephone advice line]*, there is a significant correlation with her ultimately buying* [corporation name’s] *products*” (FME).

### Segmentation and targeting

**Fig. 3:**
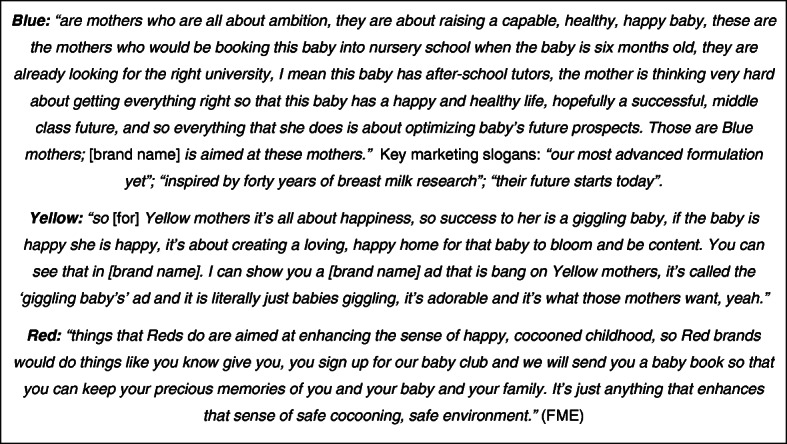
Global Market Segmentation: Blue, Yellow and Red Mothers

This type of bespoke marketing means that one size will not fit all. The personal data are therefore used to segment customers into smaller, more homogenous target groups which then receive suitably honed approaches: *“so, globally,* [corporation name] *target basically three kinds of mothers, and this is true in every country, so they call them Blue, Yellow and Red mothers* (Fig. 3) *… speaking of segmentation there’s your big three global categories”* [FME]. In western countries, for example, the market principally comprises Blue and Yellow mothers, and there is a clear difference in the type of advertising they receive. Blue mothers (and fathers) get reassuring technical claims and promises about their child’s future from *“our most advanced formulation yet”* (Fig. 3): *“‘inspired by forty years of breast milk research’, that’s a very clever claim, which essentially doesn’t mean anything technically, but which is a very clever way to imply to reduce guilt about not breastfeeding” … “That is a bang on for Blue mothers, ‘their future starts today’, she (mum) absolutely believes that”* (FME). Meanwhile, for Yellow mothers the pitch is to *“nourish their happiness”* (FME) backed up by lots of gurgling, happy babies doing endearing things. The call-outs for the baby clubs (Fig. 2) epitomise these distinctions.

Again, this is strategic: target groups do not just get their own advertisements, they get their own brands, each backed by multifaceted marketing effort. New product development, such as ‘follow-on’ milks, ‘specialist’ formulas, or, the *“BabyNes for It Moms baby nutrition system”* (an espresso-like machine which uses pods to deliver *“the exact perfect dose of milk for your baby”*) [28], keeps the category vibrant, and in the case of specialist formulas, builds useful links with the medical establishment (see below). Point of sale display in both pharmacies (for associations of quality and medical respectability) and supermarkets (for associations of value) ensures ready access. Pack design reinforces brand values and links first milks with other products. (See for example, p.35 of Harris et al. for images [29].) All of this brand support is vital because *“it’s brands that give things meaning, … it’s a short cut for communication, it bestows white powder with meaning that attracts a certain kind of woman and gets her to buy it”* (FME)*.* They give product and company a human face, a personality, a story to tell; and the sales pitch remains subtle, it is possible to “*create a brand affinity without mentioning product”* (FME).

This makes regulation extremely difficult: *“When* [corporation name] *market infant formula they do need to tiptoe a bit around stuff before 12 months* [promoting formula for babies under 12 months is supposed to be prohibited]*, but they still do all sorts of things. They don’t talk about product at all, it’s like, ‘Call our* [telephone advice line]*’, ‘Join our* [baby club]*’, no mention of a product, so you can still market without talking about a product”* (FME)*.*

### Generic effects

**Fig. 4:**
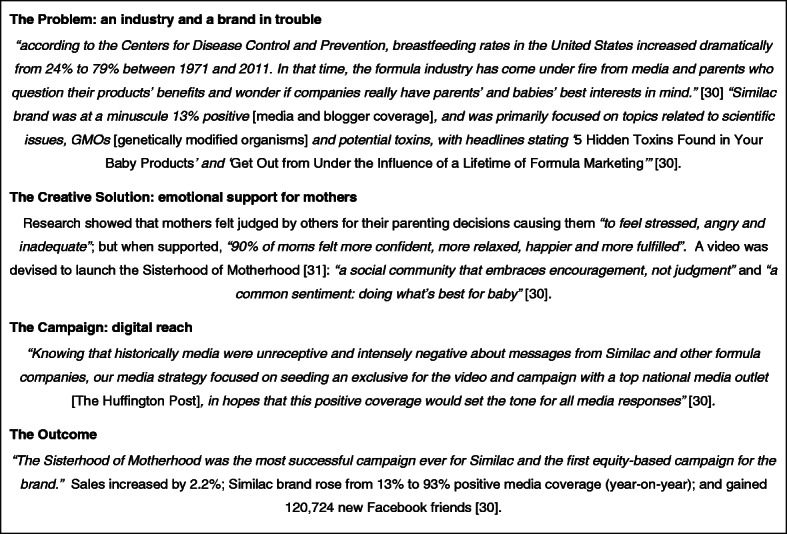
The sisterhood of motherhood [30, 31]

Formula promotion also has a generic effect, as an award-winning campaign from a US multinational demonstrates (Fig. 4). A dramatic rise in US breastfeeding rates was identified as a threat, and this was being exacerbated by negative media coverage about BMS. As a result, the brand was being undermined. An advertising agency was therefore commissioned to *“reinvigorate the Similac brand”* and also *“change the face of an entire industry”* [30]. The result was the “*Sisterhood of Motherhood*” campaign using the formula trope of *“doing what’s best for baby”* [30]. At its core is a video showing a group of parents arguing in a public park, criticising each other for their choices and differences (about nappies/careers/sexuality/gender/feeding) but when one of the buggies (strollers) runs away down the hill they stop fighting and become united in their instinct to save the baby, which, with palpable relief, they succeed in doing [31]. The strapline then appears: *“no matter what our beliefs, we are parents first – welcome to the Sisterhood of Motherhood”*. It “*was the most successful campaign ever for Similac”* resulting in increased sales and vastly improved media coverage [30]. It also succeeded in changing the narrative about infant feeding, which is no longer a matter of scientific evidence, but lifestyle choices and beliefs. Breastfeeders are positioned as just one minority, with one set of beliefs.

### Cognitive dissonance

This conceit that breast and bottle equate may help ease dissonance among formula marketers: *“everyone ‘drinks the Kool-Aid’ that it’s a good thing, it’s based on breast milk research, it’s fairly genuine from the inside. No, I don’t think anyone thinks about it as reducing rates of global breastfeeding, most people come from commercial marketing backgrounds in which you sell software, you sell sausages, oh we are selling baby food, fine …*” (FME). But, it does not entirely remove the disquiet: *“So, did I have qualms at the time? No. Would I go back to it? No, I wouldn’t”* (FME). The change of heart comes from external stimuli (in this case participating in the study played a role), not the industry: *“Nowhere is there mention of that within* [corporation name] *there is no sense that you know we are basically selling tobacco, there is no, there is no consciousness of that that I detected … I don’t think we would have worked in it if we thought we were doing something evil”* (FME).

### Targeting the medical establishment

The industry takes care to keep external stimuli supportive by building strong financial and educational links with the medical establishment: *“It creates a normality when the Royal Colleges* [two names] *that lay out the infant feeding guidelines, and set policy* [in the UK]*, and set the standard, and create leadership culture … both now have relationships with* [two corporation names]*. It sanitises it, in my view, it’s a brand sanitiser, where we have key opinion leaders, Royal Colleges, our leading paediatric institutions in terms of hospitals* [two names] *all having comfortable and cosy relationships with infant formula*” (PHE). The principal vehicle for this stakeholder marketing is ‘specialist formula’: *“The Royal College is very clear that it only accepts money for specialist formula, it doesn’t accept money for general formula. Now the* [WHO] *Code is also very clear about the marketing of breast milk substitutes, and that these specialist formulas are definitely breast milk substitutes, and they normalise interactions between industry and the profession”* (PHE). The medical profession can also provide a means of circumventing regulation, as another prize-winning campaign explains: *“Mead Johnson communicated the benefits of its Enfa A+ Gentlease baby formula directly to doctors, to work around advertising regulations in the Philippines … The approach resulted in 40% sales growth after three months*” [32].

More fundamentally, there was unease about commerce intervening in such a profoundly human area: *“the first key moment* [of pregnancy]*”, “the departure point”,* is *“really not addressed by anybody except the brands”* (FME). The search for competitive advantage can also be disturbing: *“one I find awful, in Indonesia, that they are already enriching the milk to help develop the brain even more because* [customers] *really feel that from childhood* [their child] *needs to have more, more, more; this I found very tricky … it is going too far you know?”*; *“In* [corporation name], *the next big innovation was epigenetics, so the whole idea that a baby’s success starts very early and that genes change generation to generation, so if there are stresses in the environment, that’s encoded in genes. But basically it’s saying you need to be concerned about your baby, not just from birth but from way before birth. And I think the next thing they were going to promote with* [brand name] *is that it appeals to epigenetics in some way as well, which is very Blue mother*. *That’s what they were planning; … that was the next big thing coming down the pipeline”* (FME). A 2016–17 multimedia campaign for a follow-on milk in Indonesia, is explained as addressing the “*sceptical consumer*” by launching “*the Bebelac Grow Them Great Campaign to talk about Bebelac's functionality story*”; that the formula delivers “*benefits such as* [a] *developed brain, good digestive system and well-rounded child”* [33].

### Fiscal power

#### Market size and forecast

Processed food production in the global market is dominated by a handful of powerful multinational corporations. Nestlé (US$19,370 m brand value) and Danone (US$9098 m brand value) were ranked globally by Brand Finance as the two most valuable food brands in 2018 and ranked by Kantar Worldwide in 2017 as 13th and 19th, respectively, for household reach among the leading fast-moving consumer goods brands worldwide [34]. The global dairy industry generated sales of US$204.4bn in 2017, based on Rabobank data from the 20 leading dairy corporations [34]. BMS products are just one category within a huge portfolio of products these global corporations produce.

Data from 2015 demonstrated that six multinational corporations controlled more than half of the global baby food market (including BMS), Nestlé followed by Danone holding the biggest shares, and Kraft Heinz, Mead Johnson, Abbott and FriesslandCampina the remaining four (Euromonitor International, 2015, as cited by Save the Children [18]). In 2015, Euromonitor International valued global BMS retail sales at US$47bn globally and Nestlé as the lead company accounted for 22% of these global sales [35]. Forecasting by Euromonitor for the WHO, based on the upward sales trajectory and market research to 2014, predicted that global sales would be worth US$70.7bn by 2019 [11]. Another, more conservative, estimate for investors by a multinational vegetable fats producer using 2016 Euromonitor data suggest the global retail value will be US$62.5bn by 2020, and breaks the forecast down by category: 29% standard formula (0–6 months), 21% follow-on formula (6–12 months), 43% toddler formula (> 12 months) and 7% special formula (e.g. premature and allergy) [36].

#### Marketing budgets

The marketing budget data we identified are from disparate sources and we concur with Piwoz and Huffman on the difficulty of finding open access comprehensive or verifiable data on how much money companies spend to market BMS products [14]. Overall annual advertising expenditure in 2018–19 for the two companies holding the largest portions of the global baby food and drinks market was US$944.5 m for Nestlé USA, Inc. (Glendale, CA) for national advertising expenditure that included above-the-line advertising channels plus sponsorship [37], and US$1143.3 m for Groupe Danone S.A. (Paris) for traditional media advertising, direct mail, point of purchase and product samples [38]. Another business data source described the US Mead Johnson Nutrition Company as one that markets its BMS and children’s nutrition product lines to both parents and health care professionals in Asia, Europe, Latin America and North America. The company spent $223.8 million on advertising on “*TV, print, and other consumer media, with an increasing focus on social and other direct media in 2016, up from $206.2 million in 2014*” [39].

Older Nielsen data, from 2015, breaks down advertising spend by Nestlé SA in the USA, as US$5.58 m on advertising infant formula and US$4.01 m on toddler milk [29]. Abbott spent US$3.36m advertising infant formula and US$20.71 m advertising nutritional supplements in the USA in 2015, and Mead Johnston Nutrition spent US$12.82 m advertising toddler milk and US$0.81 m on infant formula [29]. Overall, US$9.75 m were spent on advertising infant formula and US$16.83m on advertising toddler milk in the USA in 2015, mostly on television and in magazines [29].

In the UK, Nielsen data show that £13.2 m (approx. US$16.1 m) was spent on advertising BMS in 2018 using traditional media channels (which excludes sponsorship, search and social advertising channels), up 12% from the previous year but 23% lower than in 2015 [40]. BMS advertising comprised 80% of the total advertising expenditure for baby food and drinks [40]. Further analysis of Nielsen advertising data by Mintel, showed that Danone (the UK market-leader in sales value and volume) spent £13.4 m on baby food and drink (including BMS), and spent more advertising its follow-on milks (83% of spend) than other infant formula brands. As the analysts note later in their report, “*The key to growth will be in keeping older toddlers/pre-schoolers buying into the category* [baby food and drink] *for longer, if the birth rate continues to decline*”. Advertising spend by Nestlé in the UK in 2018 was far lower (less than £0.1 m), a “*dramatically reduced*” spend from the historically major spender [40].

#### Profit margins and pricing strategies

An analysis of company reports from five of the biggest baby food companies gave an indication of the profitability of the broader baby nutrition category. A 23.3% weighted average of profits demonstrates why investors are interested in the category [18, 41]. Some business analyst reports described BMS products as “*high-margin*” categories, alongside pet food and premium coffee (e.g. by Business Monitor International [42]).

Companies have taken the opportunity to premiumise their BMS products. In an investor seminar presentation, Danone endorsed their brands and strategies for mid- to long-term growth drivers in the Chinese market, including Aptamil Classic, Nutrition Classic and Aptamil Platinum, as being “*well suited to address untapped opportunity in ultra-premium IMF* [infant milk formula] *segments*”, divided into pricing segments described as “*mainstream … super premium … ultra-premium …* [and] *ultra-premium+*” [43]. Similarly, in 2017 Mead Johnson describe their ‘routine’ infant formula products and their ‘premium-priced’ product, the latter introduced into “*certain geographies*” (for the United States ‘Enspire’, and in China ‘Enfinitas’) with innovative components alleged to be “*naturally found in human breast milk* [to] *provide important benefits (lactoferrin to support immune health and MFGM* [Milk Fat Globule Membrane] *to foster cognitive development)*” [44].

### Global reach

Other global stakeholder companies include, for example, the suppliers of supplementary ingredients and packaging to the BMS industry. One US firm has agreements to supply docosahexaenoic acid to almost 30 BMS manufacturers that market products in more than 75 countries [45]. A packaging firm in Sweden, with 15 production sites in ten countries, counts the BMS industry as key customers of their “*high-performance barrier packaging solutions … to protect and promote the content*” [46].

The size and global reach are viewed by business analysts as an asset. The acquisition of Mead Johnson Nutrition Company by Reckitt Benckiser Group Plc in June 2017 “*supports future growth operations and expansion plans in various developing markets such as China, Vietnam and the Philippines and in various other parts of Latin America region*” [47]. Through Mead Johnson’s Enfamil brand (infant formula, children’s nutrition, and other nutritional products) and Nutramigen brand (specialty formula products) the company markets and sells approximately 70 different products to mothers, health care professionals, and retailers in 50 countries in Asia, North America, Latin America, and Europe [47].

Analyses funded by the WHO have estimated the carbon footprint, as greenhouse gas emissions, from the production, emissions from transport and in-home sterilisation of bottles, and preparation of powdered BMS targeted at infants of 0–6 months to be consistently higher than that for breastfeeding in all the countries tested [48]. Breastfeeding’s carbon footprint included carbon cost of the additional food required to maintain the mother’s energy balance while breastfeeding. The WHO have also cited USA data for the vast tonnage of single-use BMS packaging (plastics, cans, metal and paper) that ends up in landfills [11]. Some of the most popular BMS products in the USA are ready-to-feed single-use plastic bottles of formula with teats.

### Lobbying power

Marketing to policy makers by “*treating government departments as discrete markets to be targeted and sold to, as well as understanding the culture and buying process, …* [using] *public affairs departments … to influence government and to create good relations with them*” is part of a corporate strategy [49]. There is an imbalance of government fiscal policies in many countries that have incentivised families to use BMS rather than to breastfeed [50]. These have included government-subsidised BMS products provided through community welfare programs and BMS companies providing health workers’ education and training within countries’ hospital and public health frameworks. Further, partnerships between the BMS industry and government are increasingly proposed as solutions to infant and child food security issues [51]. Evidence of commercial stakeholders in the BMS industry influencing local policies for infant feed practices has been reported in several countries [14], and more recently in the USA, lobbying by industry stakeholders intensified before a meeting of the WHA in 2018 [52].

## Discussion

This small-scale study, in which we analyse how formula marketing and its key components work, is based on interviews with practitioners, some of whom have worked within the industry, and a review of secondary sources which detail business methods. Previous studies have described formula marketing and tried to unpick its impact on behaviour; to our knowledge ours is the first investigation to look underneath the hood and examine how the engine works. We have also assessed the size and power of the Formula Industry, to provide an indication of the resources it has at its command. The results make uncomfortable reading.

New parents are often extremely vulnerable; raising a baby is immensely challenging, and almost all parents are primarily motivated by doing the ‘best’ for their child in whatever circumstances they find themselves. They badly need reassurance and support. They also need a convenient and dependable way of feeding their child: a healthy diet compatible with hectic modern life, and the norm of working mothers and fathers. Formula companies have developed an intimate understanding of these needs and are delivering to them with a combination of ‘sympathetic’ relationship building, non-judgemental support, individually targeted communications, a readily available range of reliable products and the construction of reassuringly familiar and evocative brands. Digital marketing, where the social and commercial have melded, is greatly enhancing their efforts, whilst making the breadth of industry marketing strategies increasingly difficult to track and document.

The reach and wealth of the multinational corporation has turned this soft power into a very hard global force. The BMS market is worth about US$70bn per annum and is controlled by six of the most powerful food companies in the world, with massive household and global reach. High profit margins offer attractive investment and business opportunities. Marketing spend is extremely difficult to quantify accurately but certainly runs into billions of dollars annually, which is used to target governments and stakeholders as well as consumers. This is corporate marketing at its most powerful and disturbing.

The concerns are twofold. First, in most cases, formula feeding is not the best option, from a health or ecological standpoint. As noted above, its use is causing immense harm to babies, mothers and the environment. Second, the marketing is built on deception. Infant formula is in reality the definitive one-size-fits-all product. By law all products must have the same formulation, as established by independent research. The only permitted variation from this is for unproven additives, which if they ever prove to be beneficial, would, again by law, have to be added to all formula products. The product ranges, the segmentation and bespoke targeting, the carefully honed brands are simply subterfuge. In the UK the two leading and supposedly very different brands which dominate the market are in fact made by the same multinational.

This study was limited to a small number of interviews and relied on access to secondary data, mostly from high-income economy countries. Thus it is not representative and in particular reveals less than we would like about what is happening in the global south. Nonetheless, it provides key insights into how infant formula marketing works, and adds to our understanding of how international business impacts ill-health [53].

## Conclusions

There is an urgent need to shed more light on the harm being done by infant formula marketing; its extent is revelatory to all but a small group of public health experts. Even the marketing practitioners who had worked in the industry were taken aback by it and began to express overt regrets about their past actions. Just as formula is being normalised, so too is formula marketing. Corporate marketing careers move between companies and sectors – from formula to supermarkets to tech – this unthinking and completely unwarranted moral equivalence has to be challenged. The medical establishment has also been pulled into this charade; just as fifty years ago it had to rethink tobacco, so today it needs to review fundamentally its relationship with the formula industry. The recent decision by the BMJ and sister journals to refuse infant formula advertising is a welcome move in this direction [54].

The regulation of marketing needs to be greatly strengthened; as one marketing practitioner observed: *“the most effective response would be to prohibit any formula marketing at all; much like is done with tobacco”* (FME)*.* The point is well-made, but formula is not tobacco; it can be an essential option in specific circumstances – with preterm or SGA (small-for-gestational-age) infants, for instance, or when, even with optimal support, breastfeeding proves impossible. The problem is not the product but rather out-of-control marketing, which is driving dangerous over-consumption in the interests of corporate profits. This needs to change. The sole purpose of communications about formula should be to help parents and carers make the best possible decision for the baby. Advertising does nothing to help in this regard. It promotes spurious product differences and reinforces these with confected brands. In its digital form, which has become so prominent in recent years, it is particularly manipulative. All this advertising should cease forthwith, as demanded by the WHO Code four decades ago [16]. The pack should be unbranded and become a platform for objective guidance, from an accredited public health source, explaining the product contents, how it should be used and by whom. Point of sale activity should add further health promotion support, again from an independent source. Pricing also needs be tightly regulated; infant formula is immensely profitable for a small number of multinational corporations, while the costs to society are enormous. In addition, it should no longer be possible to use price as a bogus indicator of quality.

Only with these radical revisions will we get an infant formula market that serves the needs of babies and their parents rather than shareholders. They are big steps that will take careful, sustained management and will meet resistance from very powerful vested interests. In other contested fields, where radical change is needed, such as tobacco and climate, a Framework Convention, with its global reach, has provided the answer [55]; the equivalent is now needed for infant feeding.

## Data Availability

The datasets generated and/or analysed during the current study are not publicly available because of the need to protect participant privacy but are available from the corresponding author on reasonable request.

## Abbreviations

BMS: Breast milk substitutes
FME: Formula marketing experts
GDP: Gross domestic product
GMO: Genetically modified organisms
PHE: Public health experts
WARC: World Advertising Research Center
WHA: World Health Assembly
WHO: World Health Organization
YOY: Year on year
